# Spin-polarized Majorana zero modes in proximitized superconducting penta-silicene nanoribbons

**DOI:** 10.1038/s41598-023-44739-7

**Published:** 2023-10-20

**Authors:** R. C. Bento Ribeiro, J. H. Correa, L. S. Ricco, I. A. Shelykh, Mucio A. Continentino, A. C. Seridonio, M. Minissale, G. Le Lay, M. S. Figueira

**Affiliations:** 1 Centro Brasileiro de Pesquisas Físicas, Rua Dr. Xavier Sigaud, 150, Urca, Rio de Janeiro, RJ 22290-180 Brazil; 2 Universidad Tecnológica del Perú, Nathalio Sánchez, 125, 15046 Lima, Peru; 3 AGH University of Krakow, Academic Centre for Materials and Nanotechnology, al. A. Mickiewicza 30, 30-059 Kraków, Poland; 4 Science Institute, University of Iceland, Dunhagi-3, 107 Reykjavik, Iceland; 5 Russian Quantum Center, Skolkovo IC, Bolshoy Bulvar 30 bld. 1, Moscow, 121205 Russia; 6 School of Engineering, Department of Physics and Chemistry, São Paulo State University (UNESP), Ilha Solteira, SP 15385-000 Brazil; 7 Aix-Marseille Université, CNRS, PIIM UMR 7345, 13397 Marseille Cedex, France; 8 Instituto de Física, Universidade Federal Fluminense, Av. Litorânea s/N, Niterói, RJ CEP: 24210-340 Brazil

**Keywords:** Nanoscience and technology, Physics

## Abstract

We theoretically propose penta-silicene nanoribbons (p-SiNRs) with induced *p*-wave superconductivity as a platform for the emergence of spin-polarized Majorana zero-modes (MZMs). The model explicitly considers the key ingredients of well-known Majorana hybrid nanowire setups: Rashba spin-orbit coupling, magnetic field perpendicular to the nanoribbon plane, and first nearest neighbor hopping with *p*-wave superconducting pairing. The energy spectrum of the system, as a function of chemical potential, reveals the existence of MZMs with a well-defined spin orientation localized at the opposite ends of both the top and bottom chains of the p-SiNR, associated with well-localized and nonoverlapping wave function profiles. Well-established experimental techniques enable the fabrication of highly ordered p-SiNRs, complemented by a thin lead film on top, responsible for inducing *p*-wave superconductivity through proximity effect. Moreover, the emergence of MZMs with explicit opposite spin orientations for some set of model parameters opens a new avenue for exploring quantum computing operations, which accounts for both MZMs and spin properties, as well as for new MZMs probe devices based on spin-polarized electronic transport mechanisms.

## Introduction

Ultra-scaling of nanoelectronic devices, beyond Moore’s law, still using the ubiquitous silicon technology, could come from silicene^1–3^, the first silicon-based graphene-like artificial two-dimensional (2D) quantum material, which further engendered the Xenes family^4^, and which was used to fabricate an atom-thin channel in a field effect transistor^5, 6^. Moreover, topological silicon nanowires hosting Majorana fermions could be a materials platform for a quantum computer^7^. However, like other nanowire candidates, even proximitized ones based on heavier constituents with larger spin-orbit coupling, until now, no conclusive experimental measurements guarantee incontrovertibly the existence of topologically protected Majorana zero modes (MZMs) for the possible realization of qubits^8, 9^.

Since the appearance of the generic Kitaev model^10^, several platforms were proposed to realize it, both from theoretical^11–17^, and experimental points of view^18–24^. A helpful review of the experimental state-of-the-art on this subject can be found in Refs.^9, 25, 26^. This model considers *p*-wave superconductor pairing between electrons in different sites of a one-dimensional chain (Kitaev chain) and predicts the existence of unpaired MZMs at opposite ends of a finite Kitaev chain. However, until now, there are no conclusive experimental measurements that guarantee without doubt the existence of topologically protected MZMs^26–30^. The experimental detection of MZMs remains an elusive problem, and they were not really observed until now. Per se, this situation justifies the search for new platforms.

One possible alternative platform is the one-dimensional honeycomb nanoribbons (HNRs) that have been receiving growing attention in the literature^31–34^. Nevertheless, the mono-elemental 2D graphene-like materials coined Xenes, where X represents elements from group IIIA to group VIA of the periodic table, could constitute possible candidates to build HNRs with the ability to harbor MZMs at their ends^35–38^. Penta-Silicene (X=Si) is an up-and-coming candidate in this family for obtaining a p-SiNR geometry that can host MZMs^39–41^.

**Figure 1:**
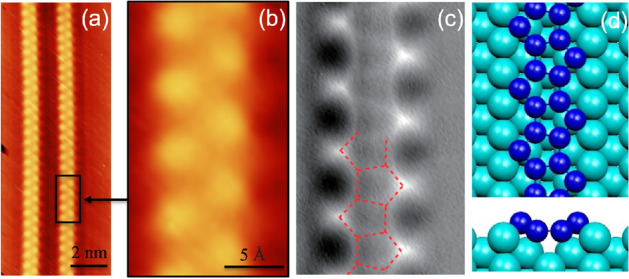
p-SiNR on Ag(110) surface. (**a**,**b**) Experimental STM images (uncorrected drift), (**c**) High-resolution nc-AFM image. (**d**) Top and cross view of the arrangement of the Si pentagonal building blocks. (**a**,**b**) Courtesy Eric Salomon, (**c**) Reprinted with permission from^40^. Copyright 2023 American Chemical Society. (**d**) From Cerda et al.^39^.

A paradigmatic breakthrough would be the experimental implementation of the generic Kitaev toy model with a silicon platform^10^. In a previous work^34^, we addressed the problem of Majorana spin discrimination employing a double-spin Kitaev zigzag honeycomb nanoribbons (KzHNR), which mimics two parallel Kitaev chains connected by the hopping *t* (see figure 1 of^34^). Since such KzHNRs have not been realized in experiments, we look instead in the present paper at the possibility of obtaining MZMs in p-SiNRs, harboring Dirac fermions, which have been epitaxially grown on Ag(110) surfaces^39, 42–44^. Typically, highly perfect, atom thin, massively aligned single strand p-SiNRs, 0.8 nm in width, and with lengths extending to tens of nanometers were obtained by molecular beam epitaxy upon in situ Si deposition onto Ag(110) surfaces held at room temperature, as shown in Fig. 1a. In scanning tunneling microscopy (STM) and high-resolution nc-AFM images, these p-SiNRs appear as two shifted lines of protrusions along the [110] direction as shown in Fig. 1b,c and are separated by twice the nearest neighbor Ag-Ag distance, i.e., 0.577 nm. Their hidden internal atomic structure was initially uncovered employing thorough density functional theory (DFT) calculations and simulations of the STM images^39^, pointing to an arrangement of pure Si pentagonal building blocks, as displayed in Fig. 1d, which defines the missing pentagonal row (P-MR) model employed in the Supplemental information of reference^39^ to optimize the angles and the distance between the silicon atoms in the pentagonal arrangement. This unique atomic geometry was later directly visualized by high-resolution non-contact atomic force microscopy (Fig. 1c from^40^). We will theoretically demonstrate that these p-SiNRs could constitute a tantalizing disruptive new Kitaev platform.

Theoretically, the most well-established example of a topological superconductor hosting MZMs is a spinless chain with *p*-wave superconducting pairing between neighboring sites, as proposed by Kitaev^10^. This type of superconductivity seems to be extremely rare in nature^45^. However, spinless orbital *p*-wave superconductivity can be engineered from a conventional spin-singlet *s*-wave superconductor when associated with a material exhibiting helical bands^46^. This was first achieved experimentally by Mourik et al.^19^, using a semiconducting nanowire with strong Rashba spin-orbit interaction in proximity to an *s*-wave superconductor subject to a magnetic field aligned along the axis of the nanowire. Despite significant advances in sample fabrication, measurement techniques, and theoretical understanding, a definitive signature of MZMs detected through electronic transport measurements in such hybrid systems remains challenging^47^. The main reason stems from inevitable inherent disorder, which generates trivial zero-energy states, mimicking the MZMs signatures^48^.

We propose a new experimental platform to circumvent this disorder issue, still within the same conceptual approach. Compared to previous proposals of Majorana nanowire devices, the paradigmatic difference is the replacement of the semiconducting nanowire by a new, highly ordered material, with transport measurements replaced by scanning tunneling spectroscopy (STS). Specifically, our proposal relies on the implementation of long and atomically precise p-SiNRs, grown in situ under ultra-high-vacuum (UHV) in the cleanest conditions on the Ag(110) surface, all aligned along the [110] direction^39–41^.

The localized zero energy states associated with MZMs at the ends of p-SINRs can be detected by low-temperature STS via an STM immersed in a strong parallel magnetic field, following the methodology of Yazdani and co-workers^49^. Since silver is not a superconductor, we will proximitize the p-SiNRs in situ with lead islands. As mentioned before, lead is a conventional BCS superconductor with a relatively high critical temperature that can be easily grown at Ag(110) surfaces^50^, which is known to interact only very weakly with the Si nanoribbons while preserving its structural and electronic properties^51, 52^. To this end, a thin lead film will be evaporated in situ on top of the p-SiNRs and annealed at temperature $\sim 200^{\circ }\text {C}$, as already mentioned in^4^. Hence, at variance with the classical fabrication and measurement procedures, all the steps in the experiments and measurements will be performed in perfectly controlled and highly cleaned conditions in a UHV system, which will comprise a surface science chamber (with all analytical tools and the two Si and Pb evaporators) directly linked to the STM/STS chamber.

Moreover, within the present proposal, we characterize the topological phase transitions (TPTs) employing the spinless version of the model and the inclusion of the *p*-wave superconducting pairing and the magnetic field reveal the emergence of topologically protected MZMs with the spin discriminated at opposite ends of the p-SiNRs; this result constitutes the main finding of the work. We also calculate the wave function of the MZMs at the ends of the p-SiNR, showing its topological signature.

**Figure 2:**
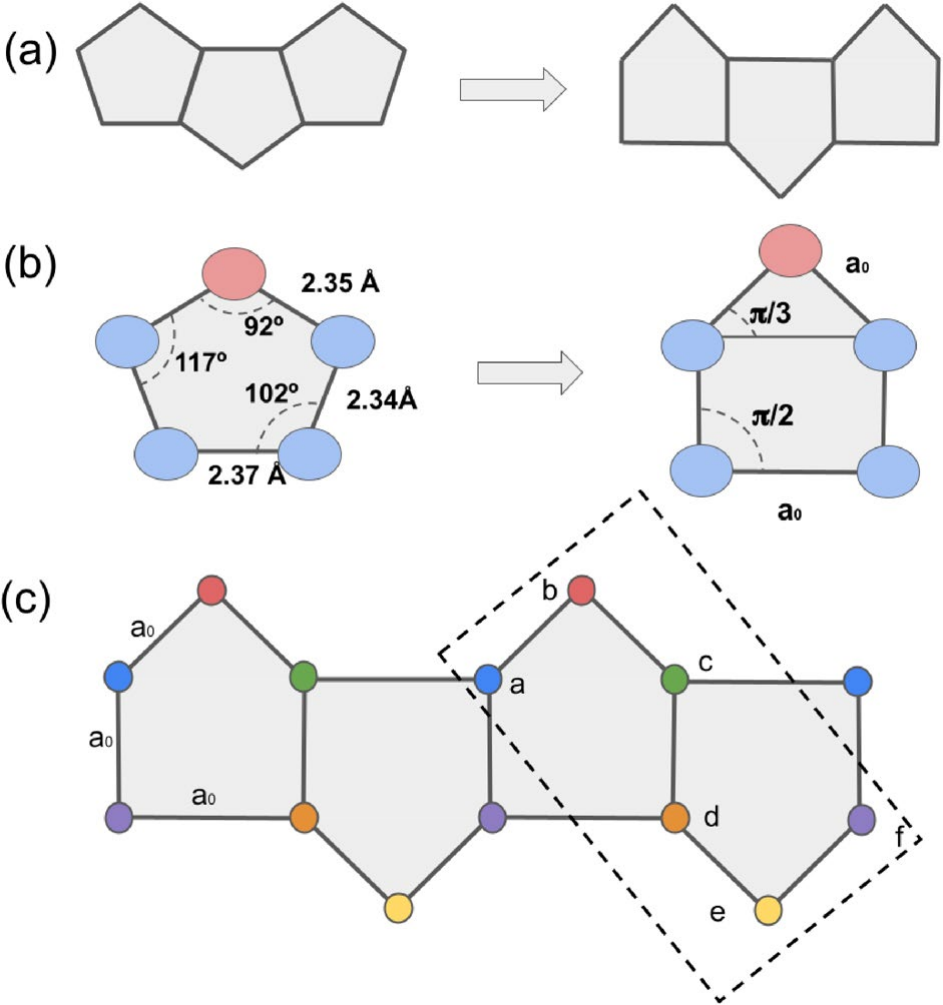
(**a**) Penta-silicene (p-SiNRs) lattice transformation adopted. (**b**) Penta-silicene angles. (**c**) Sketch of nonequivalent Si atoms placed at the vertices of the “square” pentagonal lattice. The dashed rectangle depicts the unit cell of the system.

## The model

### Lattice transformations

In Fig. 2a, to reduce the geometry complexity of the p-SiNR and facilitate the tight-binding calculations, we redefine its structure using square-shaped pentagons. In the geometry of the pentagons that constitute the p-SiNRs of Fig. 2b, four silicon atoms are located on the missing silver row, and only one exhibits a buckling structure (pink atoms). We neglect the buckling structure of these atoms and employ a planar configuration composed of square-shaped pentagons. As the distance between the silicon atoms that constitute the pentagons are close, we consider them equal to $a_{0}$ and identify it as the lattice parameter of the p-SiNR. We also define the nearest neighbor hopping as equal to *t*, which is considered the energy unit in all the calculations. $L\equiv 2Na_{0}$, is the length of the p-SiNR, and *N* is the number of sites of the corresponding Kitaev chain (top or bottom), employed in the calculation, as indicated in Fig. 2c, that exhibits the shape of the p-SiNR and the unit cell composed of six atoms inside the dashed rectangle employed in the calculations. We expect these simplifications will not change the results once we keep the lattice.

**Figure 3:**
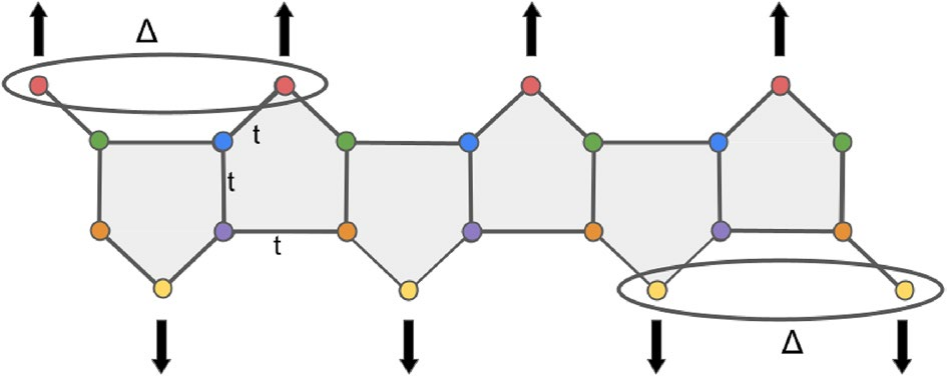
Sketch of the p-SiNRs: The penta-silicene system comprises two Kitaev chains, one on the top and the second on the bottom, hybridized via hopping *t*. The ellipses represent the superconducting *p*-wave pairing between silicon atoms belonging to the same chain, represented by pink (top) and yellow (bottom,) respectively (in the real material, these atoms correspond to the buckled one). The arrows only indicate the spin polarization needed to define a Kitaev chain.

### Effective Hamiltonian—spinless case

The total Hamiltonian, which describes the spinless p-SiNR of Fig. 3 is given by

1 $$\begin{aligned} H = H_{t} + H_{\Delta }, \end{aligned}$$

with

2 $$\begin{aligned} \begin{aligned} H_t&=- \sum _{i=1} ^{N} \mu \left( a^{\dagger }_{i, +}a_{i, +} - a^{\dagger }_{i, -}a_{i, -} +b^{\dagger }_{i, +}b_{i, +}-b^{\dagger }_{i, -}b_{i, -} + c^{\dagger }_{i, +}c_{i, +} -c^{\dagger }_{i, -}c_{i, -} \right. \\&\quad \left. + d^{\dagger }_{i, +}d_{i, +}-d^{\dagger }_{i, -}d_{i, -}+ e^{\dagger }_{i, +}e_{i, +}-e^{\dagger }_{i, -}e_{i, -} +f^{\dagger }_{i, +} f_{i, +} -f^{\dagger }_{i, -}f_{i, -} \right) \\&\quad -\sum _{i=1} ^{N} t \left( a^{\dagger }_{i, +} b_{i, +} - b_{i, -} a_{i, -}^{\dagger }+ b^{\dagger }_{i, +} c_{i, +}- c_{i, -} b_{i, -}^{\dagger } \right. \\&\quad + c^{\dagger }_{i, +} d_{i, +}- d_{i, -} c_{i, -}^{\dagger } + \left. d^{\dagger }_{i, +} e_{i, +}- e_{i, -} d_{i, -}^{\dagger } + e^{\dagger }_{i, +} f_{i, +}- f_{i, -} e_{i, -}^{\dagger } \right) \\&\quad - \sum _{i=1} ^{N-1} t\left( a^{\dagger }_{i+1, +} f_{i, +}- f_{i, -} a_{i+1, -}^{\dagger } + a^{\dagger }_{i+1, +} c_{i, +} - c_{i, -} a_{i+1, -}^{\dagger } +d^{\dagger }_{i+1, +} f_{i, +}- f_{i, -} d_{i+1,-}^{\dagger }\right) + \text {H.c.}, \end{aligned} \end{aligned}$$

where $\mu$ is the chemical potential, the index (−) and ($+$) differentiate the creation and annihilation operators for electrons and holes, respectively, and H.c. is the Hermitian conjugate. The system Hamiltonian of Eq. (2) was built according to the unit cell of nonequivalent Si atoms (a, b, c, d, e, f) shown in Fig. 2c.

The p-SiNRs are grown on Ag(110) surfaces in the setup proposed here. However, silver is not a superconductor, and to generate a *p*-wave pairing $\Delta$ on the pink and yellow atoms of Fig. 3, we evaporate in situ a thin lead film over the Ag(110) surface in such a way that the buckled silicon atoms enter in contact with the lead atoms. Under the presence of a strong Rashba spin orbit coupling (RSOC) arising from the Pb atoms and an applied magnetic field, the *s*-wave Cooper pairs of the Pb film can enter into the p-SiNR region via proximity effect (Andreev reflections)^12^, giving rise to a *p*-wave-induced pairing in the double p-SiNRs structure. By following the same procedure done in our previous work^34^ and based on the Kitaev model^10^, we introduce a spinless *p*-wave superconducting pairing $\Delta$ between the “external” pink and yellow atoms of the same type as shown in Fig. 3. The Hamiltonian, which describes such a pairing, reads

3 $$\begin{aligned} \begin{aligned} H_{\Delta }= \sum _{i=1} ^{N-1} \Delta \left( b^{\dagger }_{i,+} b^{\dagger }_{i+1,-} - b^{\dagger }_{i+1,-} b^{\dagger }_{i,+} +e^{\dagger }_{i,+} e^{\dagger }_{i+1,-} - e^{\dagger }_{i+1,-} e^{\dagger }_{i,+} \right) + \text {H.c.}. \end{aligned} \end{aligned}$$

### Effective Hamiltonian—spinful case

In order to properly account for the spin degree of freedom in the superconducting p-SiNRs, we follow our previous work^34^. Considering also the spin degree of freedom on both $H_t$ and $H_{\Delta }$,

4 $$\begin{aligned} H_t= & {} -\sum _{i=1, \sigma }^{N} \mu \Bigl ( a^{\dagger }_{i, +, \sigma }a_{i, +, \sigma } - a^{\dagger }_{i, -, \sigma }a_{i, -, \sigma } + b^{\dagger }_{i, +, \sigma }b_{i, +, \sigma }- b^{\dagger }_{i, -, \sigma }b_{i, -, \sigma }+ c^{\dagger }_{i, +, \sigma }c_{i, +, \sigma } - c^{\dagger }_{i, -, \sigma }c_{i, -, \sigma } \nonumber \\{} & {} + d^{\dagger }_{i, +, \sigma }d_{i, +, \sigma } - d^{\dagger }_{i, -, \sigma }d_{i, -, \sigma } + e^{\dagger }_{i, +, \sigma }e_{i, +, \sigma } - e^{\dagger }_{i, -, \sigma }e_{i, -, \sigma } + f^{\dagger }_{i, +, \sigma }f_{i, +, \sigma } - f^{\dagger }_{i, -, \sigma }f_{i, -, \sigma } \Bigr )\nonumber \\{} & {} -\sum _{i=1,\sigma }^{N} t \Bigl ( a^{\dagger }_{i, +, \sigma } b_{i, +, \sigma } - b_{i, -, \sigma } a_{i, -, \sigma }^{\dagger } + b^{\dagger }_{i, +, \sigma } c_{i, +, \sigma } - c_{i, -, \sigma } b_{i, -, \sigma }^{\dagger } + c^{\dagger }_{i, +, \sigma } d_{i, +, \sigma } \nonumber \\{} & {} - d_{i, -, \sigma } c_{i, -, \sigma }^{\dagger } + d^{\dagger }_{i, +, \sigma } e_{i, +, \sigma } - e_{i, -, \sigma } d_{i, -, \sigma }^{\dagger } + e^{\dagger }_{i, +, \sigma } f_{i, +, \sigma } - f_{i, -, \sigma } e_{i, -, \sigma }^{\dagger } \Bigr ) \nonumber \\{} & {} -\sum _{i=1,\sigma }^{N-1} t \Bigl ( a^{\dagger }_{i+1, +, \sigma } f_{i, +, \sigma } - f_{i, -, \sigma } a_{i+1, -, \sigma }^{\dagger } + a^{\dagger }_{i+1, +, \sigma } c_{i, +, \sigma } - c_{i, -, \sigma } a_{i+1, -, \sigma }^{\dagger } \nonumber \\{} & {} + d^{\dagger }_{i+1, +, \sigma } f_{i, +, \sigma } - f_{i, -, \sigma } d_{i+1, -, \sigma }^{\dagger } \Bigr ) + \text {H.c.} \end{aligned}$$

5 $$\begin{aligned} H_{\Delta }= & {} \sum _{i=1,\sigma } ^{N-1} \Delta \left( b^{\dagger }_{i,+, \sigma } b^{\dagger }_{i+1,-, \sigma } - b^{\dagger }_{i+1,-, \sigma } b^{\dagger }_{i,+, \sigma } + e^{\dagger }_{i,+, \sigma } e^{\dagger }_{i+1,-, \sigma } - e^{\dagger }_{i+1,-, \sigma } e^{\dagger }_{i,+, \sigma } \right) + \text {H.c.}. \end{aligned}$$

We introduce a Zeeman effect due to the application of an external magnetic field perpendicular to the p-SiNRs plane. The Hamiltonian, which accounts for the Zeeman effect, reads:

6 $$\begin{aligned} \begin{aligned} H_z&= \sum _{i=1, \sigma }^{N} Z \ sgn(\sigma ) \left( a_ {i,\sigma } ^{\dagger } a_{i,\sigma } + b_{i,\sigma } ^{\dagger } b_{i,\sigma } + c_{i,\sigma } ^{\dagger } c_{i,\sigma } + d_{i,\sigma } ^{\dagger } d_{i,\sigma } + e_{i,\sigma } ^{\dagger } e_{i,\sigma } + f_{i,\sigma } ^{\dagger } f_{i,\sigma } \right) + \text {H.c.}, \end{aligned} \end{aligned}$$

wherein $Z\,$ is the effective strength of the external Zeeman magnetic field $\vec {B}$, and $\sigma =\uparrow ,\downarrow$ is the spin index for each operator.

The extrinsic RSOC induced in the p-SiNRs can be modulated by the action of an external electric field $\vec {E}$ applied perpendicularly to the nanoribbon plane^53–56^. Its corresponding general Hamiltonian reads

7 $$\begin{aligned} H_R= \sum _{i,j=1,\sigma }^{N} i c_{i, \sigma } ^{\dagger } (\vec {u}_{i,j}. \vec {\gamma }) c_{j, ({\bar{\sigma }})} + \text {H.c.}, \end{aligned}$$

where $\vec {u}_{i,j} = -\frac{R \ }{a_0} {\hat{k}} \times \vec {\delta }_{i,j}$, with $R\,$ being the extrinsic RSOC parameter, $\vec {\delta }_{i,j}$ is the vector that connects the adjacent lattice sites *i* and *j*, and $\vec {\gamma }$ the Pauli matrices. The index ${\bar{\sigma }}$ indicates the opposite spin direction of $\sigma$. The Eq. (7) turns into

8 $$\begin{aligned} \begin{aligned} H_R&= \sum _{i=1,\sigma }^{N} \left( \gamma _{1}( a^{\dagger }_{i, \sigma } b_{i+1/2, {\bar{\sigma }}} ) + \gamma _{2} ( b^{\dagger }_{i+1/2, \sigma } a_{i, {\bar{\sigma }}}) \right. \\&\quad + (a^{\dagger }_{i, \sigma } c_{i-1, {\bar{\sigma }}} ) - (c^{\dagger }_{i-1, \sigma } a_{i, {\bar{\sigma }}}) + (-i) ( a^{\dagger }_{i, \sigma } f_{i, {\bar{\sigma }}} ) \\&\quad +(i) (f^{\dagger }_{i, \sigma } a_{i, {\bar{\sigma }}}) + \gamma _{3} (b^{\dagger }_{i+1/2, \sigma } c_{i+1, {\bar{\sigma }}}) + \gamma _{4} (c^{\dagger }_{i+1, \sigma } b_{i+1/2, {\bar{\sigma }}}) \\&\quad + (-i)(c^{\dagger }_{i+1, \sigma } d_{i+1, {\bar{\sigma }}}) + (i)(d^{\dagger }_{i+1, \sigma } c_{i+1, {\bar{\sigma }}}) -(d^{\dagger }_{i+1, \sigma } f_{i, {\bar{\sigma }}}) \\&\quad + (f^{\dagger }_{i, \sigma } d_{i+1, {\bar{\sigma }}}) + \gamma _{3} (d^{\dagger }_{i+1, \sigma }e_{i+3/2, {\bar{\sigma }}}) + \gamma _{4} (e^{\dagger }_{i+3/2,\sigma } d_{i+1, {\bar{\sigma }}})\\&\quad + \left. \gamma _{1} (e^{\dagger }_{i+3/2,\sigma }f_{i+2, {\bar{\sigma }} }) + \gamma _{2} (f^{\dagger }_{i+2,\sigma } e_{i+3/2, {\bar{\sigma }}}) \right) + \text {H.c.}, \end{aligned} \end{aligned}$$

where $$\gamma _{1} = \frac{-1}{2} \ + \frac{i\sqrt{3}}{2}$$ , $$\gamma _{2} =\frac{1}{2} \ - \frac{i\sqrt{3}}{2}$$ , $$\gamma _{3} =\frac{-1}{2} \ - \frac{i\sqrt{3}}{2}$$ and $$\gamma _{4} =\frac{1}{2} \ + \frac{i\sqrt{3}}{2}$$.

Notice that from Eqs. (6) and (7), we are assuming the external Zeeman magnetic field $\vec {B}$ perfectly perpendicular to the RSOC, i.e, $B\equiv B_{\perp }\ne 0$ and $B_{\parallel } = 0$. In Rashba nanowires setups, this condition is responsible for the vanishing of the induced superconducting gap at zero momentum (inner gap) and the opening of a constant gap at finite momentum (outer gap), which characterizes the topological phase transition and the concomitant emergence of MZMs protected by the outer gap^12^.

However, from the experimental perspective, ensuring that the magnetic field is applied only in the perpendicular direction of the RSOC field can be challenging. Then, it is natural to consider also the effects of $B_{\parallel } \ne 0$. In this situation, we have both components of the Zeeman field, and the critical magnetic field condition for the topological phase transition remains the same. However, the behavior of the outer gap is not constant anymore, which affects the topological protection of the MZMs towards fault-tolerant quantum computing operations. The effect of $B_{\parallel }$ in the outer gap is not so detrimental if the RSOC is strong.

It is worth noticing that the opposite cases of $B \equiv B_{\parallel } \ne 0$ and $B_{\perp } = 0$ can lead to the vanishing of the outer gap, hence preventing the topological phase and emergence of MZMs. Therefore, since our system is qualitatively described by the similar underlying physics of Rashba nanowires, it is appropriate to experimentally ensure the dominance of the magnetic field component perpendicular to the Rashba field.

We now can define the total system Hamiltonian as

9 $$\begin{aligned} H_{\text {total}} = H_t + H_Z + H_R + H_{\Delta }, \end{aligned}$$

which can be written in the corresponding Bogolyubov–de Gennes (BdG) form in *k*-space as $H_{\text {total}}(k)=\Phi ^{T}{\varvec{H}}_{\text {BdG}}(k)\Phi$, with

10 $$\begin{aligned} \begin{aligned} {\varvec{H}}_{\text {BdG}}(k) = \displaystyle \left[ \begin{matrix} H_{\uparrow , \uparrow }(k)&{}\quad H_{\uparrow , \downarrow }(k) &{}\quad H_{\Delta ,\uparrow , \uparrow }(k) &{}\quad H_{\Delta ,\uparrow , \downarrow }(k)\\ H_{\uparrow , \downarrow }(k)&{}\quad H_{\downarrow , \downarrow }(k) &{}\quad H_{\Delta ,\downarrow \uparrow }(k) &{}\quad H_{\Delta ,\downarrow , \downarrow }(k) \\ H^* _{\Delta ,\uparrow ,\uparrow }(-k) &{}\quad H^* _{\Delta ,\uparrow ,\downarrow }(-k) &{}\quad H^*_{\uparrow , \uparrow }(-k) &{}\quad H^*_{\uparrow , \downarrow }(-k) \\ H^* _{\Delta ,\downarrow ,\uparrow }(-k) &{}\quad H^* _{\Delta ,\downarrow ,\downarrow }(-k) &{}\quad H^*_{\downarrow , \uparrow }(-k) &{}\quad H^*_{\downarrow , \downarrow }(-k) \end{matrix}\right] , \displaystyle \end{aligned} \end{aligned}$$

where $H_{\sigma ,\sigma '}(\pm k)$ and $H_{\Delta ,\sigma ,\sigma '}(\pm k)$ represent the matrix elements for different spin directions and the matrix elements corresponding to the part of the matrix where superconducting couplings $\Delta$ appear, respectively. The spinor $\Phi$ was constructed with the fermionic operators in the following order:

11 $$\begin{aligned} \begin{aligned} \Phi ^{T}&=(a_{k,\uparrow },b_{k,\uparrow },c_{k,\uparrow },d_{k,\uparrow },e_{k,\uparrow },f_{k,\uparrow },a_{k,\downarrow },b_{k,\downarrow },c_{k,\downarrow },d_{k,\downarrow },e_{k,\downarrow },f_{k,\downarrow }, \\ {}&\quad a^{\dagger }_{-k,\uparrow },b^{\dagger }_{-k,\uparrow },c^{\dagger }_{-k,\uparrow },d^{\dagger }_{-k,\uparrow },e^{\dagger }_{-k,\uparrow },f^{\dagger }_{-k,\uparrow }, a^{\dagger }_{-k,\downarrow },b^{\dagger }_{-k,\downarrow },c^{\dagger }_{-k,\downarrow },d^{\dagger }_{-k,\downarrow },e^{\dagger }_{-k,\downarrow },f^{\dagger }_{-k,\downarrow }). \end{aligned} \end{aligned}$$

The spin alignment for each situation in the next section is computed numerically. We calculate the mean value of the Pauli matrix in ${\hat{z}}$ direction ${\hat{S}}_z$, i.e., $\langle {\hat{S}}_z \rangle = \langle \Psi |{\hat{S}}_z|\Psi \rangle$, where $|\Psi \rangle$ are the eigenvectors of the total Hamiltonian given by Eq. (9).

In hybrid semiconducting-superconducting nanowires, sometimes called Majorana nanowires, the following features strongly suggest the emergence of MZMs at the nanowire ends^12^:

- Closing and subsequent reopening of the superconducting gap in the bulk relation dispersion as the chemical potential $\mu$ changes, indicating a TPT;
- Emergence of persistent zero-modes for specific system parameter values associated with nonoverlapping wave functions localized at the opposite ends of the nanowire.

To obtain the TPTs present in the p-SiNRs, we will consider the infinite case given by the Hamiltonian of Eq. (1). We calculate the bulk band structure, discussed in detail in the supplemental material (SM). To investigate the existence of MZMs in the p-SiNRs, we will analyze the spinless p-SiNRs with finite size $N=100$ and calculate the energy spectrum as a function of the chemical potential $\mu$ and the probability density function $|\psi |^2$ associated with the zero-energy states which arise on the real axis of the energy spectrum.

Both the energies $E_{n}$ and eigenvectors $\psi _{n}$ per site are obtained by numerically solving the Schrödinger equation $H \psi _n = E_n \psi _n$ for the Hamiltonian of Eq. (1). To evaluate the position dependence of the wave functions associated with zero energy states, we numerically calculate the eigenvector $\psi _{n}$ when $E_{n}=0$, which allows obtaining the probability density per lattice site according to

12 $$\begin{aligned} | \psi _n | ^2 = \psi _n \psi _n ^{*}. \end{aligned}$$

**Figure 4:**
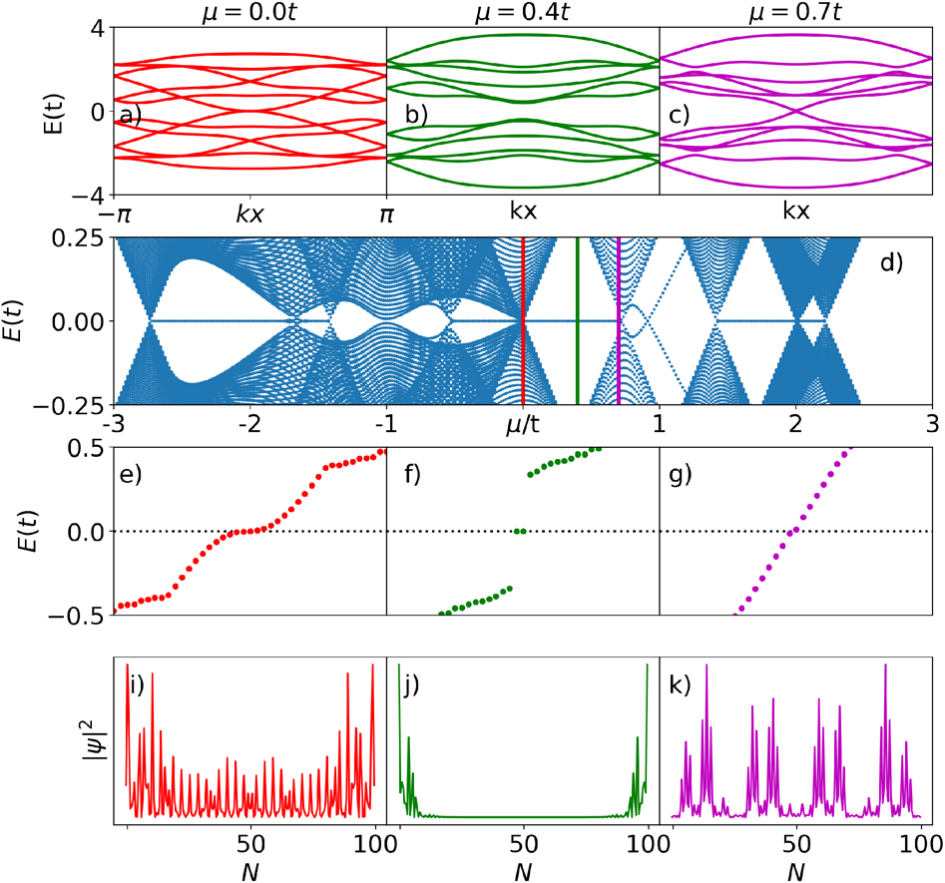
Spinless case: (**a**–**c**) Bulk energy dispersion for the spinless p-SiNRs as a $k_{x}$ function. The colors red, green, and magenta used in the panels correspond to $\mu =0.0t$, 0.4*t*, and 0.7*t*, respectively. (**d**) Energy spectrum as a function of the chemical potential. (**e**–**g**) Zero-energy states spectrum. (**i**–**k**) Probability density per lattice site $|\psi |^2$, associated with zero-energy states on the real axis of the Kitaev, top or bottom chains.

## Results and discussion

### Finite spinless p-SiNRs

We employed the following parameter set in all the calculations: $\Delta = 0.5 t$, $Z=0.1t$, $R=0.05t$ and $N=100$. The top panels of Fig. 4 show the bulk energy dispersion of the p-SiNRs, in the presence of the superconducting *p*-wave pairing, described by Eqs. (1–3), along the $k_{x}$ direction, for three representative values of chemical potential $\mu$ [vertical lines in panel (d)]. Figure 4a depicts the closing of the superconductor (SC) gap at $k_{x}=0$ for $\mu = 0.0t$. As the value of $\mu$ enhances, the SC gap opens as shown in panels (b) for $\mu = 0.4t$ and closes again at $k_{x}=0$ for $\mu = 0.7t$ as shown in panel (c). This closing and reopening of the SC gap with the tuning of $\mu$ characterize a topological phase transition. The bulk-boundary correspondence principle^57^ ensures the topologically protected MZMs at the ends of the p-SiNRs.

To verify the emergence of MZMs associated with the TPTs depicted in Fig. 4a–c, we plot the p-SiNRs energy spectrum as a function of $\mu$ in Fig. 4d. There are no zero-energy modes for the values of $\mu$ where the gap closes (red and magenta vertical lines). However, for values of $\mu$ inside the topological gap, for example, when $\mu =0.4t$ (green vertical line), two zero-energy states appear on the real axis, indicating the presence of MZMs at the opposite ends of the p-SiNRs, topologically protected by the effective *p*-wave SC gap (Fig. 4b). This finding is similar to what was obtained in our previous work^34^, wherein the MZMs emerge at the opposite ends of a finite double zHNR.

Figure 4f shows isolated zero-energy modes for $\mu =0.4t$, which are associated with a nonoverlapping wave function, well-localized at the ends of the p-SiNRs, as depicted in Fig. 4j; which together with the topological phase transition (Fig. 4a–c), is a piece of strong evidence that topologically protected MZMs emerge at the opposite ends of the spinless p-SiNRs. In the Supplemental Material, we developed an extensive analysis of the topological and trivial phases of the spinless *p*-wave superconducting p-SiNR, that can be distinguished by the Zak number topological invariant^58^. However, we cannot afford to do the same study for the spinful case due to the extreme mathematical complexity.

Although there are zero-energy modes for other values of $\mu$ (Fig. 4e,g), they are not associated with wave functions well-localized at the ends of the p-SiNR, as can be seen in Fig. 4i,k, for $\mu =0.0t$ and $\mu =0.7t$, respectively. This implies that a zero mode cannot be exclusively attributed to MZMs. In this context, the genuine nature of the zero-modes shown in Fig. 4e,f, for instance, can be experimentally distinguished by combining STM and atomic force microscopic (AFM) measurements^49, 59^. This approach allows associating the zero-bias conductance peaks at the p-SINR ends (Fig. 4f) with their well-localized wave functions through spatially resolved conductance maps, cf. Fig. 4j.

Furthermore, we highlight that we analyze only one region of all energy spectrum shown in Fig. 4d, which presents other ranges of chemical potentials wherein a zero-energy state, associated with the emergence of MZMs, arises. A more detailed study of the energy spectra can be found in the SM. We can also observe that, unlike the system of our previous work^34^, the energy spectrum of Fig. 4d is asymmetric at about $\mu = 0$.

**Figure 5:**
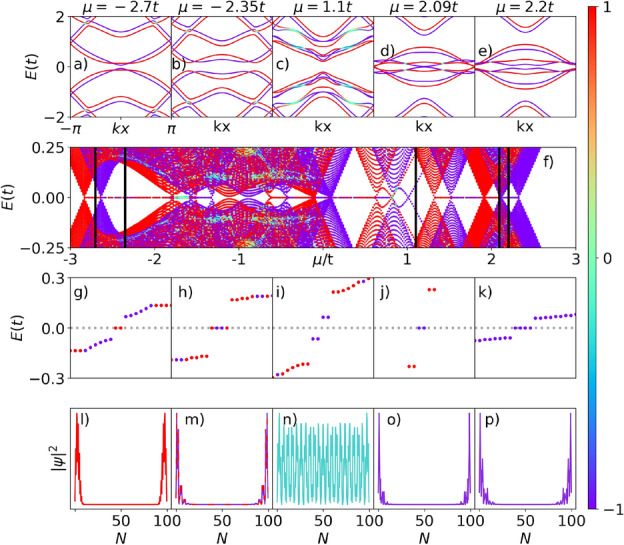
Spinful case—magnetic field up: (**a**–**e**) Bulk energy dispersion of the superconducting p-SiNRs for the spinful situation, as a function of $k_{x}$, for $\mu =-2.7t$, $-2.35t$, 1.1*t*, 2.09*t* and 2.2*t*, respectively. (**f**) Energy spectrum as a function of the chemical potential. Vertical lines indicate the chosen values of chemical potential shown on top panels. (**g**)–(**k**) Zero-energy states spectrum. (**l**)–(**q**) Probability density per lattice site $|\psi |^2$, associated with zero-energy states on the real axis of the spin-polarized Kitaev, top or bottom chains.

**Figure 6:**
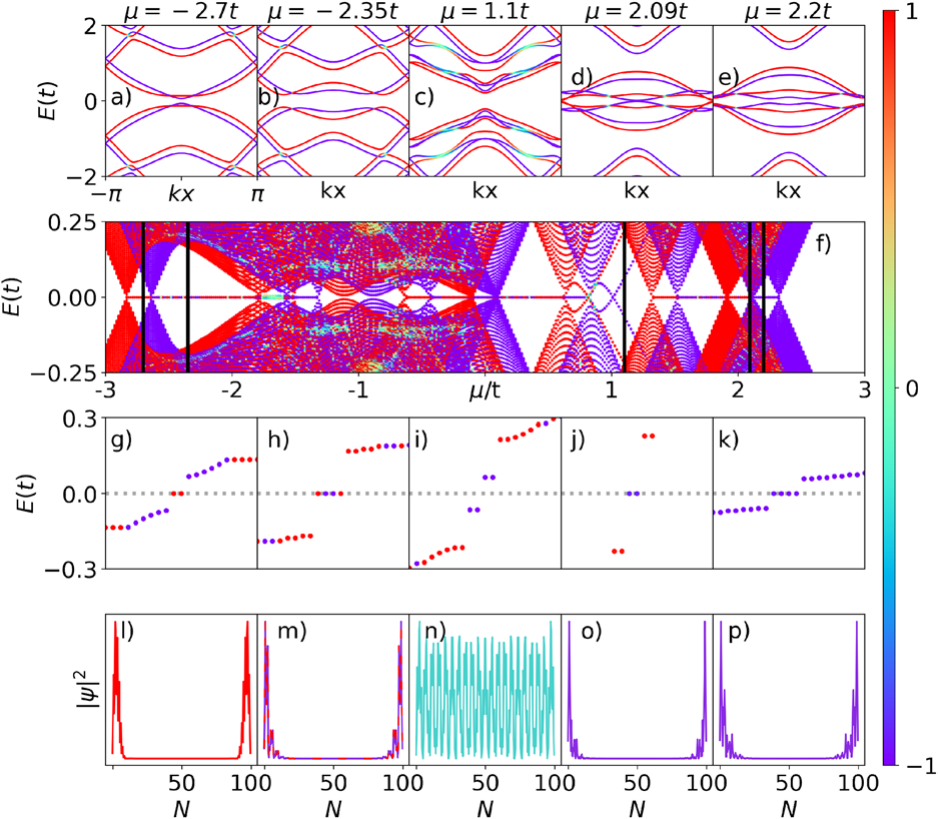
Spinful case—magnetic field down: The same situation of Fig. 5 but with the magnetic field pointing in the opposite direction.

### Finite spinful p-SiNRs

Now we will analyze how the spinless scenario shown in Fig. 4 is affected by the presence of both Zeeman field (Eq. 6) and extrinsic RSOC (Eq. 7) coupling within the spinful description (Eq. 9).

Figure 5a–e exhibit the energy dispersion of the p-SiNRs given by the eigenenergies of BdG Hamiltonian (Eq. 10) as a function of $k_{x}$, for distinct values of the chemical potential $\mu$, indicated by vertical lines in Fig. 5f. The spin polarization is indicated by the vertical color bar, in which the red color represents the spin $\uparrow =1$, while the blue color stands for spin $\downarrow =-1$, and the light shades of colors mean the spin is neither up nor down. As $\mu$ is tuned, we can see the opening and closing of the superconducting gap, thus indicating a TPT, as previously verified in the spinless situation (Fig. 4a–c). However, here we can notice that each TPT associated with a specific value of $\mu$ has a preferential spin orientation, except Fig. 5c, where the system exhibits a conventional band gap.

The spin-polarized TPTs in Fig. 5a,b,d,e lead to the appearance of spin-polarized zero-modes in Fig. 5f, which shows the system energy spectrum as a function of $\mu$. These zero-modes indicate the emergence of spin-polarized MZMs at the ends of the p-SiNRs as $\mu$ is changed, similar to those found in^34^.

The panels g–k of Fig. 5 depict the corresponding energy levels sorted in ascending order. The different values of $\mu$ used to calculate the MZMs are indicated by vertical black lines in Fig. 5f. For $\mu =-2.7t$ (Fig. 5g), there are two zero modes on the real axis of spin up (red points), associated with nonoverlapping wave functions shown in Fig. 5l.

For $\mu =-2.35t$ (Figs. 5h and 6h), there are two energy-states in the spin-up direction and the other two with spin-down, associated with degenerate (blue and red) nonoverlapping wave functions shown in Figs. 5m and 6m, respectively. It is worth mentioning that this situation is absent from our previous paper^34^ and constitutes a pivotal difference exclusively related to the p-SiNRs. Here, pairs of MZMs at opposite edges split into top and bottom chains of the nanoribbon with opposite spins. These MZMs, acting as an effective two-level electronic system, would allow the recovery of the spin degree of freedom as a good quantum number for purposes of quantum computing, as well as to define the intrinsic spins of the regular fermions built up by these top and bottom pairs of MZMs edge states, respectively. By considering two p-SiNRs as source and drain reservoirs with an immersed quantum dot, a type of single electron transistor (SET) could be set^15, 60, 61^, allowing to detect a spin-polarized zero-bias conductance provided by one of the spins associated with a given pair of MZMs placed at one particular nanoribbon chain.

The chemical potential $\mu =1.1t$ (Fig. 5i) represents a non-topological region, there are four spin-down energy states outside the real axis, there are no MZMs, and the wave functions completely overlap along the ribbon. The system exhibits a particular “semiconductor” phase with the gap controlled by the $\mu$ variation and two delta peaks generated by the zero modes outside the real axis, inside the gap.

For $\mu =2.09t$ (Fig. 5j), there are two zero modes on the real axis of spin up (red points). Finally, for $\mu =2.2t$ (Fig. 5k), there are four MZMs with spin-down energy states on the real axis. This situation happens because, at $\mu =2.09t$, a TPT occurs for spin-up, the gap closes at $k=\pm \pi$, and for $\mu >2.09t$ the gap defines a trivial band insulator for this spin orientation and MZMs with spin-up are not available anymore. These well-localized probability densities describing wave functions centered at the opposite ends of the superconducting p-SiNRs, associated with zero-energy edge states, indicate the emergence of MZMs in the same way previously found for the spinless system.

Figure 6 represents the same situation as Fig. 5 but with the magnetic field pointing in the opposite direction. The net effect on the p-SiNRs is to change the MZMs, for all $\mu$ values, in spin up to down and vice versa. Therefore, it is possible to select the spin polarization of the MZMs by changing the chemical potential $\mu$ or the magnetic field orientation.

**Figure 7:**
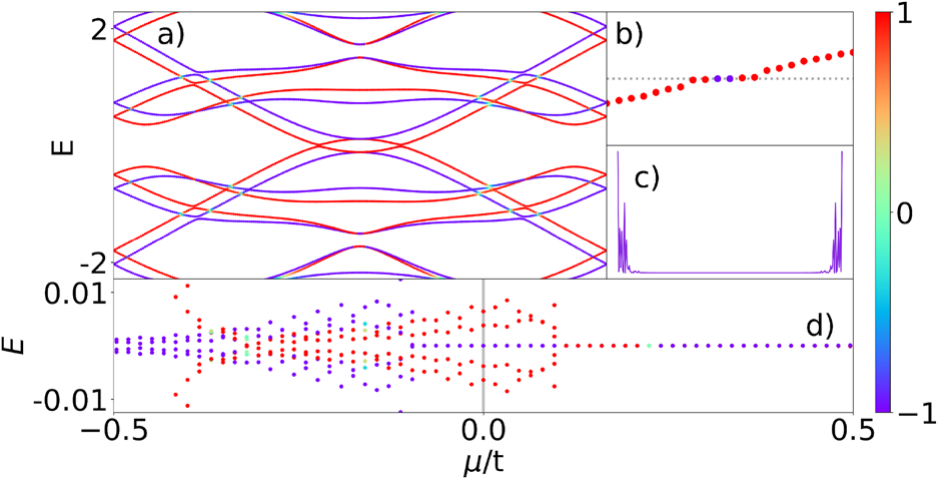
Analysis in detail of the $\mu =0$ case of Fig. 5 with the magnetic field pointing in the up direction.

**Figure 8:**
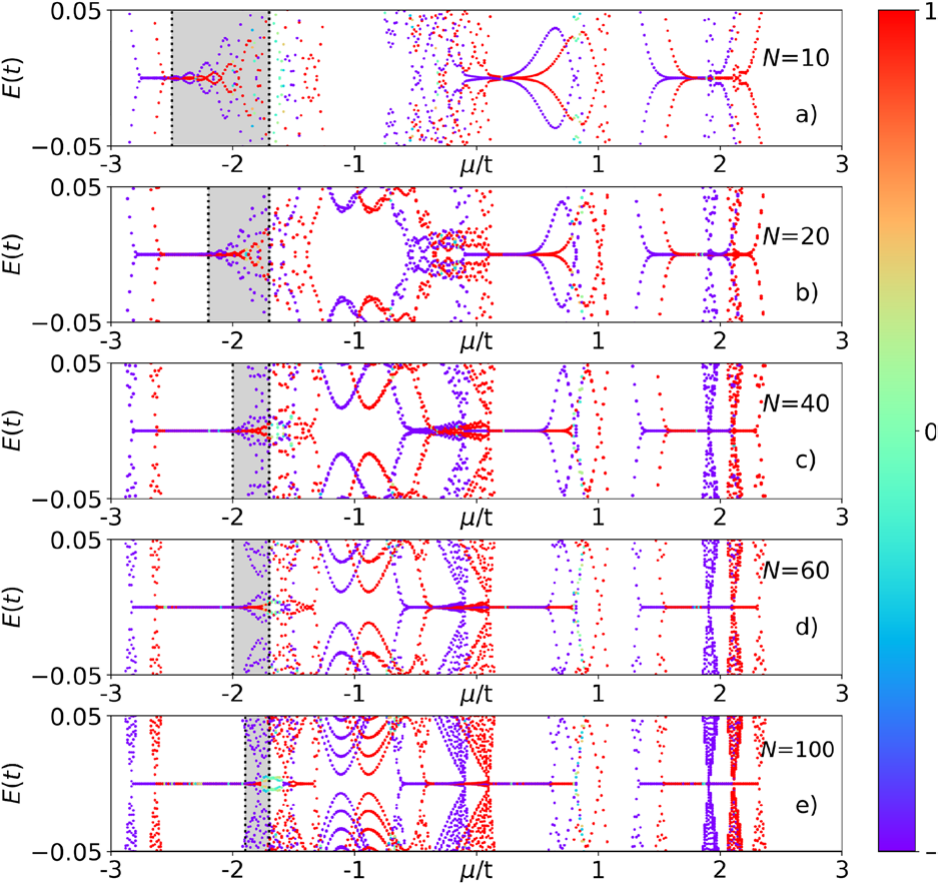
Energy spectrum as a function of the chemical potential $\mu$, for distinct lengths of superconducting p-SiNRs, namely, for $N=10$ (**a**), $N=20$ (**b**), $N=40$ (**c**), $N=60$ (**d**) and $N=100$ (**e**).

In Fig. 7, we mainly analyze the dispersion relation, energy spectrum, and nature of the zero-modes at $\mu =0$ of Fig. 5, with the magnetic field pointing in the up direction. Figure 7a depicts *E*(*k*) as a function of $k_x$, showing that there is a finite topological superconducting gap only for the spin-down orientation (blue line), while the spin-up (red line) remains gapless. This behavior suggests a spin-polarized TPT at zero chemical potential, implying that only the system’s spin-down component is within the topological regime. At the same time, the spin-up belongs to a metallic phase. Fig. 7b,c represent two MZMs of spin-down with its correspondent nonoverlapped wave function, respectively, and Fig. 7d, shows detail at around $\mu =0$ region.

Additionally, we investigate how the energy spectrum as a function of $\mu$ is affected by the length of the p-SiNRs. Fig. 8 exhibits the superconducting p-SiNRs’ energy spectrum for increasing nanoribbon length values *N*. From the smallest system considered ($N=10$, Fig. 8a) to the largest one ($N=100$, Fig. 8e), it can be noticed a decrease of the amplitude of oscillations at around the real axis ($E=0$), and at the same time the definition of the MZMs on the real axis improves as *N* increases, and for $N=100$ the MZMs are well defined in all the real axis. It should be mentioned that these oscillations around zero energy are expected for short Majorana nanowires due to the overlap between Majorana wavefunctions of opposite ends. Therefore, such oscillations are expected to decrease as the system becomes larger. The same behavior was verified in the work^34^.

## Conclusions and perspectives

This paper demonstrates the emergence of topologically protected MZMs at opposite ends of spinless and spinful p-SiNRs with *p*-wave superconducting pairing. These MZMs exhibit spin discrimination, and their polarization can be controlled by adjusting the nanoribbon chemical potential or the external magnetic field. To implement our findings experimentally, we propose a material engineering of p-SiNRs grown over an Ag(110) surface (cf. Fig. 1a), with a thin Pb film deposited on top^4, 50^. In this device, the proximity effect will enable the penetration of Cooper pairs from the Pb *s*-wave superconductor into the p-SiNRs^12^, and in combination with an external magnetic field and the extrinsic RSOC modulated by the action of an external electric field $\vec {E}$ applied perpendicularly to the nanoribbon plane^53–56^, it will induce *p*-wave pairing in the buckled atoms of the double p-SiNRs structure (cf. Fig. 1d).

We should highlight the potential applications driven by the spin-polarized MZMs presented in this work, notably demonstrated in the results of Fig. 7, with the down spin component associated with MZMs, while the up component displays metallic features, resulting in a half-metallic behavior for the system^62, 63^. This property could be harnessed to design a single Majorana transistor (SMT) built from a quantum dot (QD) sandwiched by finite p-SiNR leads^6, 64, 65^. This setup resembles the conventional single electron transistor (SET)^66^. The SMT can be a valuable tool for discerning between MZMs and trivial Andreev bound states^15, 17^. Particularly, the leakage of MZMs through the QD^67^, along with both local and crossed Andreev reflections induced by a specific spin orientation within the p-SiNR-QD-p-SiNR SMT structure, is expected to generate distinct electronic transport signatures, enabling the identification of MZMs.

In addition to the spin-polarization of MZMs, our proposal also features the emergence of two MZMs located at opposite ends of the p-SiNR top chain, while another two with opposed spins are at the bottom, as illustrated in Figs. 5h and 6h. These MZMs acting as an effective two-level electronic system would allow the recovery of the spin degree of freedom as a good quantum number for purposes of quantum computing implementation, as well as to define the intrinsic spins of the regular fermions built-up by these top and bottom couples of edge MZMs, respectively. It is crucial for implementing quantum computing operations between two qubits, as it requires the presence of two fermionic sites, i.e., four MZMs^68, 69^. Therefore, our proposal is a promising candidate for realizing hybrid quantum computing operations^70, 71^ between conventional qubits and spin-polarized Majorana-based qubits and paves the way for defining quantum computing operations using Majorana spintronics^72^.

### Supplementary Information

Supplementary Information.

## Data Availability

The data that support the findings of this study are available from the corresponding author upon reasonable request.
