# Supplementary material for: Spin-polarized Majorana zero modes in proximitized superconducting penta-silicene nanoribbons
Source: Sci Rep. 2023 Oct 20;13:17965. doi: 10.1038/s41598-023-44739-7 (PMC10589331; doi:10.1038/s41598-023-44739-7)
Supplement: Supplementary file 1 — Supplementary Information. [file 41598_2023_44739_MOESM1_ESM.pdf]

Supplementary Material: Spin-Polarized Majorana Zero Modes in Proximitized Superconducting Penta-Silicene Nanoribbons

R. C. Bento Ribeiro,[1] J. H. Correa,[2,3] L. S. Ricco,[4] I. A. Shelykh,[4,5] Mucio A. Continentino,[1] A. C. Seridonio,[6] M. Minissale,[7] G. Le Lay,[7] and M. S. Figueira[8]

[1]*Centro Brasileiro de Pesquisas Físicas, Rua Dr. Xavier Sigaud, 150, Urca 22290-180, Rio de Janeiro, RJ, Brazil*

[2]*Universidad Tecnológica del Perú, Nathalio Sánchez, 125, 15046, Lima, Perú*

[3]*AGH University of Krakow, Academic Centre for Materials and Nanotechnology, al. A. Mickiewicza 30, 30-059 Krakow, Poland*

[4]*Science Institute, University of Iceland, Dunhagi-3, IS-107,Reykjavik, Iceland*

[5]*Russian Quantum Center, Skolkovo IC, Bolshoy Bulvar 30 bld. 1, Moscow 121205, Russia*

[6]*School of Engineering, Department of Physics and Chemistry, São Paulo State University (UNESP), 15385-000 Ilha Solteira-SP, Brazil*

[7]*Aix-Marseille Université, CNRS, PIIM UMR 7345, 13397 Marseille Cedex, France*

[8]*Instituto de Física, Universidade Federal Fluminense, Av. Litorânea s/N, CEP: 24210-340, Niterói, RJ, Brasil*

(Dated: October 4, 2023)

I. TOPOLOGICAL CLASSIFICATION AND ZAK PHASE TOPOLOGICAL INVARIANT

The classification of the topological phases of matter is provided by the analysis of fundamental symmetries for a given Hamiltonian in the discrete reciprocal space [1–3], namely time-reversal ($\mathcal{TR}$), particle-hole ($\mathcal{PH}$) or charge conjugation and chiral symmetries ($\mathcal{K}$).

For the particular case of the spinless penta-silicene nanoribbons (p-SiNRs) with p-wave superconducting pairing at their edges [Eq. (1-3) of main text], it is verified that both $\mathcal{TR}$ and $\mathcal{PH}$ symmetries are preserved, once

$$\mathcal{T}h(k)\mathcal{T}^{-1} = h(-k) \tag{1}$$

and

$$\mathcal{C}h(k)\mathcal{C}^{-1} = -h(-k), \tag{2}$$

where $\mathcal{T}$ and $\mathcal{C}$ are the time-reversal and charge conjugation operators, respectively, and $h(k)$ is a matrix coming from the Hamiltonian of Eq. (1-3) in the manuscript, rewritten in the Bogoliubov-de Gennes (BdG) representation, i.e.,

$$\mathcal{H}(k) = \frac{1}{2}\sum_{k} \Psi_k^{\dagger} h(k) \Psi_k, \tag{3}$$

with

$$\Psi_k \equiv (a_k, a_{-k}^{\dagger}, b_k, b_{-k}^{\dagger}, c_k, c_{-k}^{\dagger}, d_k, d_{-k}^{\dagger}, e_k, e_{-k}^{\dagger}, f_k, f_{-k}^{\dagger})^T \tag{4}$$

being the spinor, which accounts the assumption of $\mathcal{PH}$ symmetry.

The fulfilment of both $\mathcal{TR}$ and $\mathcal{PH}$ symmetries directly implies that the $\mathcal{K}$ symmetry is also preserved [3], meaning that

$$\mathcal{K}h(k)\mathcal{K}^{-1} = -h(k), \tag{5}$$

where $\mathcal{K} = \mathcal{T} \cdot \mathcal{C}$ corresponds to the chiral operator. Moreover, from the relations expressed in Eqs. (1), (2) and (5), we obtain $\mathcal{T}^2 = 1$, $\mathcal{C}^2 = 1$ and $\mathcal{K}^2 = 1$, meaning that the BdG Hamiltonian [Eq. (3)] of the spinless superconducting p-SiNR [Eq. (1-3), main text] is a representative of the BDI symmetry class [3], the same class of the well-known Kitaev chain [4].

It is worth mentioning that spinless superconducting p-SiNR is a simplification considering an "intrinsic" magnetic field. The presence of this field is crucial for inducing the

formation of p-wave superconducting pairing along the nanoribbon edges. However, in practical experimental setups, the source of the spin-polarization is an external magnetic field that naturally breaks the $\mathcal{TR}$ symmetry and hence, $\mathcal{K}$ symmetry. From this argument, the "artificial" $\mathcal{TR}$ symmetry of the spinless model can be neglected. Thus the BdG Hamiltonian of Eq. (3) belongs to the D symmetry class [1, 3]. Therefore, the p-SiNR in presence of an applied magnetic field is a $\mathbb{Z}_2$ superconductor in one-dimension [3, 5], once the proposed double-spin Kitaev zigzag nanoribbon configuration can be regarded as two interconnected Kitaev chains with a hopping term (cf. discussion in the main text).

From the previously discussed perspective, the topological and trivial phases of the spinless p-wave superconducting p-SiNR, as described by the BdG Hamiltonian of Eq. (3), can be distinguished by the Zak number topological invariant [6]

$$\varphi_{\mathrm{Zak}} = -\int_{-\pi}^{\pi} \frac{dk}{2\pi i} \partial_k \ln\left[\mathrm{Det}(A(k))\right]. \tag{6}$$

A nonzero quantized Zak phase φ_{Zak} is associated with the emergence of topologically protected edge states, which is an outcome of the conventional bulk-boundary correspondence [2, 3]. Specifically, the integer values of φ_{Zak} topological invariant correspond to the number of topologically protected edge modes present in the system and characterize its topological phase transitions (TPTs).

To compute the Zak number through Eq. (6), it is necessary to obtain a chiral matrix $\mathcal{A}(k)$ associated with $h(k)$, which is performed through the computation of a unitary transformation outlined below:

$$\tilde{h}(k) = \mathcal{U}^{\dagger} h(k) \mathcal{U} = \begin{bmatrix} 0 & A(k) \\ A^{*}(k) & 0 \end{bmatrix}, \tag{7}$$

bringing $h(k)$ to its chiral form, where

$$A(k) = \begin{bmatrix} 2\mu & 2te^{\frac{ik}{2}} & 2te^{-ik} & 0 & 0 & 2t \\ 2te^{-\frac{ik}{2}} & 2\Phi_k + 2\mu & 2te^{\frac{ik}{2}} & 0 & 0 & 0 \\ 2te^{ik} & 2te^{-\frac{ik}{2}} & 2\mu & 2t & 0 & 0 \\ 0 & 0 & 2t & 2\mu & 2te^{\frac{ik}{2}} & -2te^{-ik} \\ 0 & 0 & 0 & 2te^{-\frac{ik}{2}} & 2\Phi_k + 2\mu & 2te^{\frac{ik}{2}} \\ 2t & 0 & 0 & -2te^{ik} & 2te^{-\frac{ik}{2}} & 2\mu \end{bmatrix}, \tag{8}$$

is the chiral matrix, with $\Phi_k = i\Delta \sin(2k)$.

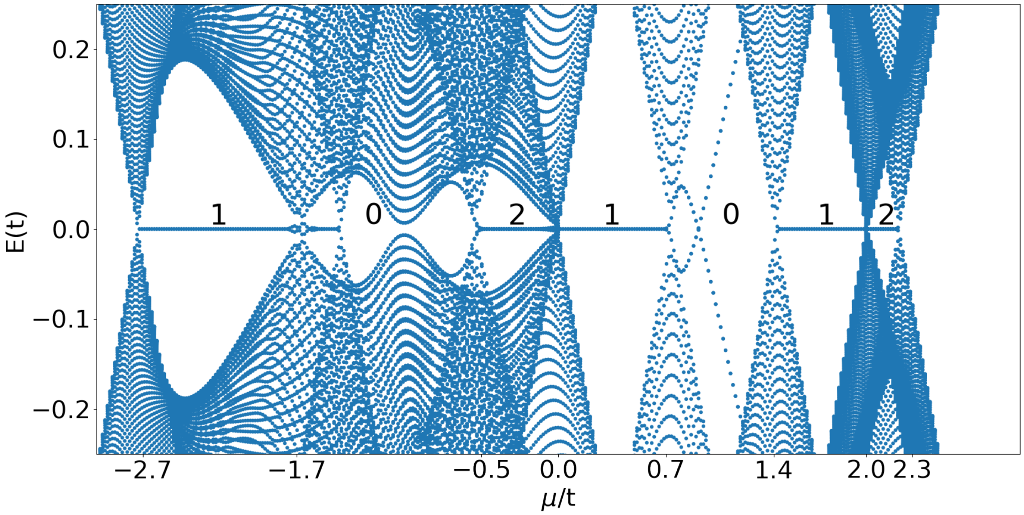

FIG. 1. Energy dispersion of the bulk system as a function of the chemical potential μ, for the spinless p-SiNR with p-wave superconducting pairing between the atoms localized at the edges, cf. Eq. (1) of the main text. The Zak phase φ_{Zak}, represented by the values 0, 1, and 2, corresponds to the number of MZMs present at the edges of either one or both chains comprising the p-SiNR.

By considering Eq. (8) and Eq. (6) and employing numerical integration, it becomes feasible to compute the Zak number for several values of chemical potential μ. The manipulation of μ triggers the closing and subsequent reopening of the superconducting gap, a phenomenon closely related to the TPTs, as discussed in the main text.

In this context, Fig. 1 illustrates the Zak number across distinct regions in the bulk energy dispersion of the spinless p-wave superconducting p-SiNR. Notably, a Zak phase of zero corresponds to regions where zero modes are absent, indicating that the system resides within the topologically trivial phase. Conversely, for $\varphi_{\mathrm{Zak}} \neq 0$, zero-energy modes emerge, indicating the presence of topologically protected Majorana zero modes (MZMs) at the edges of either one ($\varphi_{\mathrm{Zak}} = 1$) or both top/bottom chains ($\varphi_{\mathrm{Zak}} = 2$) of the p-SiNR [7].

---

[1] B. A. Bernevig and T. L. Hughes, *Topological Insulators and Topological Superconductors*, STU - Student edition ed. (Princeton University Press, 2013).

[2] C.-K. Chiu, J. C. Y. Teo, A. P. Schnyder, and S. Ryu, Rev. Mod. Phys. **88**, 035005 (2016).

[3] T. D. Stanescu, *Introduction to Topological Quantum Matter & Quantum Computation*, 1st ed. (CRC Press, 2016).

[4] A. Y. Kitaev, Physics-Uspekhi **44**, 131 (2001).

[5] R. Aguado, Riv Nuovo Cimento **40**, 523 (2017).

[6] J. Zak, Phys. Rev. Lett. **62**, 2747 (1989).

[7] R. C. B. Ribeiro, J. H. Correa, L. S. Ricco, A. C. Seridonio, and M. S. Figueira, Phys. Rev. B **105**, 205115 (2022).
